# Rare Earth Doped ZnO Nanoparticles as Spintronics and Photo Catalyst for Degradation of Pollutants

**DOI:** 10.3390/molecules28062838

**Published:** 2023-03-21

**Authors:** Pooja Dhiman, Garima Rana, Amit Kumar, Elmuez A. Dawi, Gaurav Sharma

**Affiliations:** 1 International Research Centre of Nanotechnology for Himalayan Sustainability (IRCNHS), Shoolini University, Solan 173229, India; 2 Nonlinear Dynamics Research Centre (NDRC), College of Humanities and Science, Ajman University, Ajman P.O. Box 346, United Arab Emirates

**Keywords:** ZnO, photocatalysis, tetracycline, chemical water pollutants, nanomaterials

## Abstract

Antibiotic water contamination is a growing environmental problem in the present day. As a result, water treatment is required for its reduction and elimination. Due to their important role in resolving this issue, photocatalysts have drawn a great deal of interest over the past few decades. When non-biodegradable organic matter is present in polluted water, the photo catalytic process, which is both environmentally friendly and an improved oxidation method, can be an effective means of remediation. In this regard, we report the successful synthesis of pure phased rare earth doped ZnO nanoparticles for tetracycline degradation. The prepared catalysts were systematically characterized for structural, optical, and magnetic properties. The optical band gap was tailored by rare earth doping, with redshift for Sm and Dy doped nanoparticles and blueshift for Nd doped ZnO nanoparticles. The analysis of photoluminescence spectra revealed information about the defect chemistry of all synthesised nanoparticles. Magnetic studies revealed that all synthesized diluted magnetic semiconductors exhibit room temperature ferromagnetism and can be employed for spintronic applications. Moreover, Dy doped ZnO nanoparticles were found to exhibit a maximum degradation efficiency of 74.19% for tetracycline (TCN) removal. The synthesized catalysts were also employed for the degradation of Malachite green (MG), and Crystal violet (CV) dyes. The maximum degradation efficiency achieved was 97.18% for MG and 98% for CV for Dy doped ZnO nanoparticles. The degradation mechanism involved has been discussed in view of the reactive species determined from scavenging experiments.

## 1. Introduction

Dyes and pharmaceutical pollutants are becoming the most serious commercial organic contaminants due to their poisonous and carcinogenic nature. Both people and drinking water are vulnerable to the harmful effects of organic contaminants. The pharmaceuticals, especially ‘antibiotics’, due to their increased global consumption, have raised a universal concern, as their excessive usage is polluting the water sources used for both human and agricultural purposes. Among antibiotics, tetracycline (TC) is the most commonly used antibiotic worldwide. The fundamental structural constituent of TC is a tetracyclic ring structure comprising hydroxyl, methyl, keto, and the dimethylamino functional groups. Despite the number of health benefits of TC, it is still considered a pollutant, as it enters water sources through humans or animal excreta. TC is reportedly found in domestic wastewater and hospital wastewater in evident concentrations of 1 µg L^−1^ and 100 µg L^−1^, respectively [1,2]. The elimination of organic pollutants (drugs + dyes) from the discharge of industrial wastewater, hospital wastewater, and domestic wastewater has received recognition in the field of environmental remediation. Organic dyes are carbon-containing complex compounds enriched with aromatic rings, and their nitro, sulfo, chloro, and ami-docyanogen series are particularly prevalent in industrial effluents, which are the primary source of water contamination. Color effluents from dyes and colour industries are not only causing colour problems in water bodies, but they are also becoming a source of pollution for living organisms by reducing or even stopping light and water re-oxygenation capacities, resulting in an interruption in aquatic life. These dyes have the potential to deplete dissolved O_2_, as well as to be hazardous to plants, wildlife, and humans. Methylene blue [3], crystal violet [4], malachite green [5], rhodamine-B [6], and other [7] dyes are among those that are considered harmful pollutants when they are found in water systems above the permitted limits and pollute the water sources. Thus, the invention of efficient methods to manage water contamination is currently considered a major issue in this field of study. Different methods have been implemented to eliminate the organic contaminants, including chemical oxidants, membrane processes, adsorption, and flotation. Nevertheless, the majority of the aforementioned procedures generate dangerous by-products and necessitate costly treatment methods. On the basis of this perspective, photocatalysis has garnered a significant amount of interest in the sector of environmental remediation due to the utilization of solar light, which is both a constant non-degrading source and also capable of degrading organic contaminants without producing any carcinogenic by-products [8,9,10]. Semiconductor photo catalysts are commonly used to transform light into chemical energy. The non-toxic characteristics of ZnO, combined with its features such as its economy and large surface area, have contributed to its being recognised as a leading candidate for a catalyst [11]. It is amazing that ZnO is a robust photocatalyst and is vulnerable to photocorrosion, supporting its recyclability [12]. However, pure ZnO suffers from poor quantum yield. This is because photogenerated electron-hole pairs recombine very quickly, which limits their application in photocatalysis [13]. In particular, doping other metal ions into the crystal system creates the intermediate band structure below the conduction band of ZnO, which helps to harvest a larger amount of the solar spectrum. In particular, the catalytic performance of ZnO can be boosted by doping it with metal ions, which results in defects being created in the crystal structure. In addition, metal ions can play the role of electron-trapping agents, preventing electron-hole recombination and favouring charge separation. The photocatalytic reactions can be enhanced by adding metal ions to the ZnO matrix [14,15]. Recently, it was reported that ZnO nanoparticles synthesized through precipitation method using sodium dodecyl sulfate can degrade 49% of tetracycline [16]. Similarly, the Ni doping in ZnO matrix decreased the recombination rate of the charge carriers and resulted in enhanced photodegradation efficiency (94%) for the removal of methylene blue dye [17]. A growing number of researchers are looking to rare earth metals as a dopant to improve photo catalysts for the decomposition of organic contaminants [18,19]. Rare earth enhances photocatalytic efficacy while decreasing electron-hole recombination. Rare-earth metals have an imperfect 4f orbital configuration, which helps to corral the electron, which minimises the recombination of charge carriers as well as increasing the catalytic performance [20]. In particular, the 4f electron configuration and multi-electron organization of rare-earth metals provide high thermal resistance to the ZnO structure. Vaiano et al. [21] modified ZnO with praseodymium (Pr) and observed exceptional photocatalytic performance under the exposure of ultraviolet and visible light. Zong et al. and Korake et al. investigated the excellent photocatalytic performance of eu-doped ZnO nanoparticles [22,23]. Okte et al. described the synthesis of several rare earth (La, Eu, Gd, Dy, and Ho)-loaded ZnOs and investigated their photocatalytic performance in the removal of methyl orange dye [24]. The photocatalytic efficiency of Ln (La, Nd, or Sm)-doped zinc oxide nanoparticles in the decomposition of p-nitrophenol was investigated by Khatamian et al. [18]. However, Nb doped ZnO (0.25 g/L) has the capacity to degrade 75.7% of TC (15 mg/L) in 150 min of experiment. The observed results were explained in terms of greater surface area and a narrow band gap in Nd doped ZnO catalysts [25]. Researchers mainly interested in photocatalysis for the removal of waste water may consider the involvement of rare earth metals doped into ZnO to be relevant to the presented findings. ZnO nanoparticles doped with rare-earth metal ions still need to be explored for optimal photocatalytic efficiency for the degradation of organic contaminants. Moreover, the induction of a few percent of rare earth elements into a ZnO matrix can induce room temperature magnetism, which is the required condition for spintronic applications [26,27]. However, the cause of room temperature magnetism is still controversial and needs to be explored. Both photo catalytic degradation and spintronic applications of ZnO rely directly on the synthesis route which may optimize the crystallite size, lattice defects, morphology, band gap, and magnetic properties. Rare earth doping into the ZnO structure has emerged as an effective way to modify these properties, especially defects chemistry, which plays a crucial role in determining the efficacy of the catalyst. In the present work, we have successfully synthesized rare earth doped ZnO nanoparticles, characterised them for structural, optical, and magnetic properties, and used them to degrade the tetracycline drug under UV-visible light irradiation, inspired by the potential of rare earth doping into ZnO for optimising the properties and applications in wastewater treatment. For the purposes of comparison, the malachite green and crystal violet dyes have also been tested for degradation.

## 2. Results and Discussions

### 2.1. X-ray Diffraction Analysis

**Figure 1:**
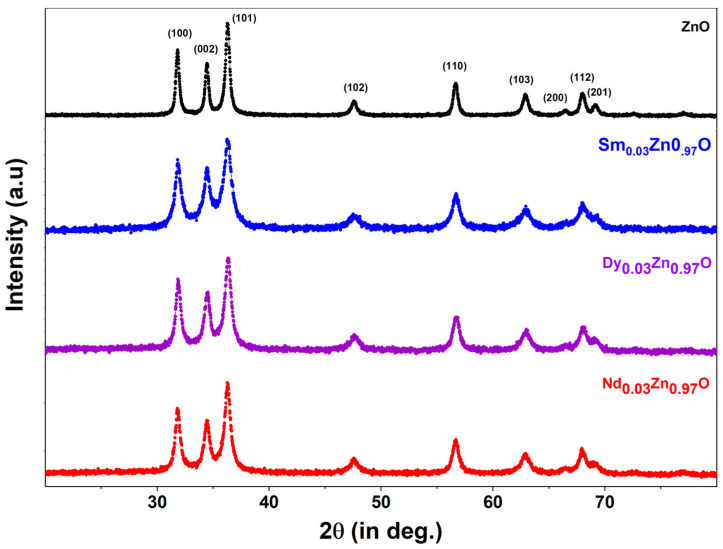
XRD pattern for undoped ZnO and doped ZnO nanoparticles.

Figure 1 demonstrates the X-ray diffraction pattern for all synthesized samples. ZnO and doped nanoparticles exhibit a pure phased hexagonal structure with indexed peaks of (100), (002), (101), (102), (110), (103), (200), (112), and (201). There is no impurity peak present in all of the prepared samples. The indexed peaks match the JCPDS card number 36-1451. There are no additional peaks related to dopants or impurities, showing that the samples are very pure and of a crystalline nature. Because of the inclusion of rare earth-metals in ZnO, the XRD patterns of all rare earth-doped ZnO indicated a trend towards lower theta values compared to pure ZnO nanoparticles. The enlargement of the unit cell caused by the mismatch in ionic radii of Zn ions and rare earth ions may be the source of the diffraction peak’s shift towards a lower theta value. Additionally, the slight shift is an indication that rare earth ions have been incorporated into the ZnO matrix. In the literature, similar findings are observed where metals create analogous alterations in the crystal structure [28]. The crystallite size of all nanoparticles was calculated using the well-known Scherrer’s equation, which is given by:

(1) $$D=\frac{k\lambda }{\beta \cos\theta }$$

where *k* is the shape factor (0.89), *D* is the crystalline size, *λ*-wavelength of the X-ray radiation (0.1542 nm), *β* is broadening of diffraction line measured at half of its maximum intensity, and *θ* is the Bragg’s angle in radians. For ZnO, Sm-ZnO, Nd-ZnO and Dy-ZnO, the *D* values determined were 19.17 nm, 12.04 nm, 13.46 nm and 13.12 nm, respectively. The other calculated structural parameters are shown in Table 1.

The following Equation (2) for the hexagonal crystal structure was used to obtain the lattice parameters for X_1-Y_Zn_Y_O (X = Sm, Dy and Nd, Y = 0.97) powders. These values were derived from the (101) and (002) planes by using the following equation:

(2) $${\left(\frac{1}{d_{hkl}}\right)}^{2}=\frac{4}{3}\left(\frac{h^{2}+k^{2}+hk}{a^{2}}\right)+\left(\frac{l^{2}}{c^{2}}\right)$$

When rare earth elements were introduced into ZnO, the *a* and *c* lattice parameters experienced a reduction in value. Similar behaviour has been observed previously, and it can be attributed to two main phenomena: the compressive hydrostatic pressure generated by rare earth dopants on the ZnO surface, and the movement of rare earth atoms imprisoned in the non-equilibrium state towards an equilibrium position [29].

**Figure 2:**
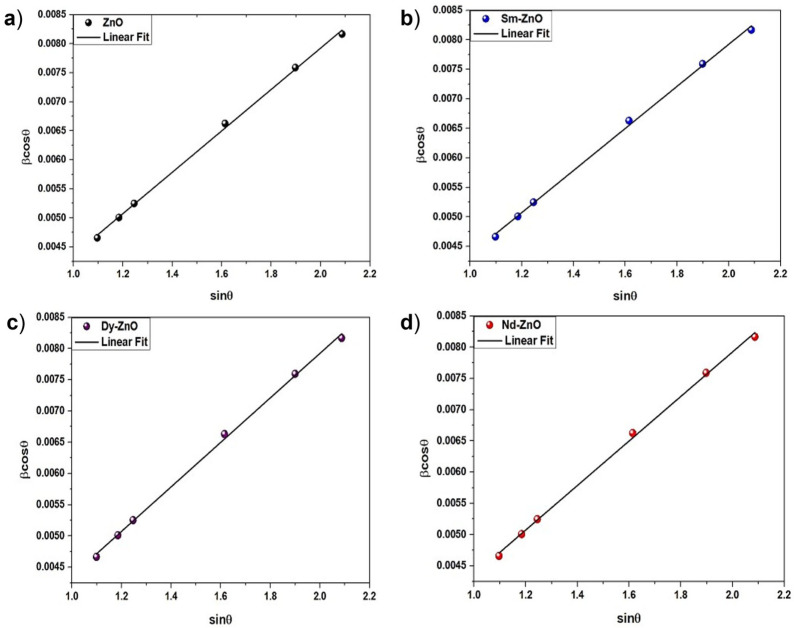
W-H plot for (**a**) ZnO nanoparticles, (**b**) Sm-doped ZnO nanoparticles, (**c**) Dy-doped ZnO nanoparticles, (**d**) Nd-doped ZnO nanoparticles.

The Williamson-Hall method was used to analyse the strain, and the following formula was used [30]:

(3) $$\beta \cos\theta =\frac{\lambda k}{D}+4\varepsilon \sin\theta$$

where *k* is the shape factor (0.89), *D* is the crystalline size, *λ* is the wavelength of the X-ray radiation (0.1542 nm), *β* is broadening of diffraction line measured at half of its maximum intensity, *θ* is the Bragg’s angle in radians, and *ε* is the effective strain. Figure 2 shows the W-H graph for the synthesized nanoparticles.

W-H plot differentiates the size and strain contribution in peak broadening which also includes instrumental broadening. In observed W-H plot, the less scattering of data points occurs due to negligible 2*θ* variations on rare earth doping at Zn sites. Additionally, the better fit of data points indicates the minimum and uniform lattice strain in samples (Supplementary Materials).

In addition to the W-H analysis, another structural parameter was studied to verify the rare earth influence on the ZnO nanoparticles. Variability in the Zn-O bond length, the degree of deformation, and the volume of the unit cell were all determined using the lattice parameters a and c, and their values were then calculated using the following equations:

(4) $$V=\frac{\sqrt{3a^{2}c}}{2}$$

(5) $$R\;=\frac{2a{\left(\frac{2}{3}\right)}^{0.5}}{c}$$

(6) $$L=\frac{\sqrt{a^{2}}}{3}+\left(0.5-d^{2}\right)$$

A perfect stoichiometric wurtzite structure is represented by the packing factors (*c*/*a*) (1.6333). The presence of oxygen and zinc vacancies is shown by a deviation from a distortion degree of 1 (*R*, where *R* = 1 signifies no evident distortion). Every sample contained evidence of oxygen and zinc vacancies, as measured by *R* values. The unit cell volume decreased as a result of the decrease in the lattice parameter. The results are shown in Table 1.

### 2.2. Surface Morphology and Particle Size Analysis

**Figure 3:**
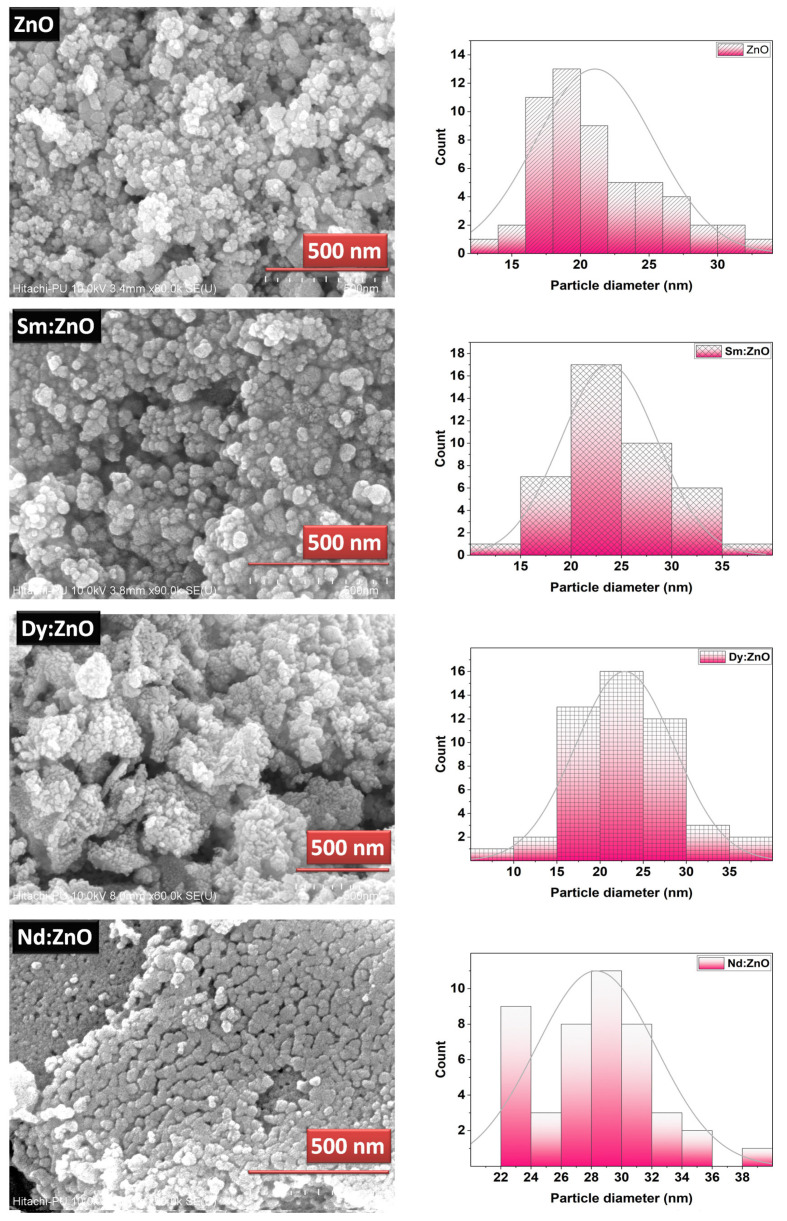
SEM images and corresponding grain size distribution plots for synthesized nanoparticles.

**Figure 4:**
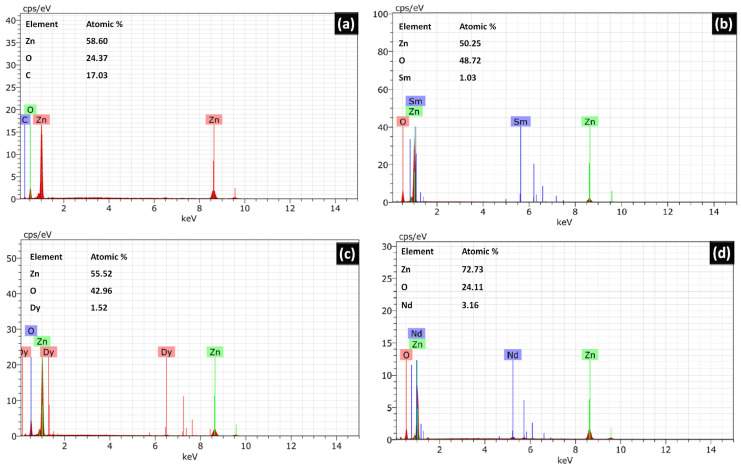
(**a**–**d**) Energy dispersive X-ray spectrum for undoped ZnO, Sm doped ZnO, Dy doped ZnO, and Nd doped ZnO nanoparticles.

**Figure 5:**
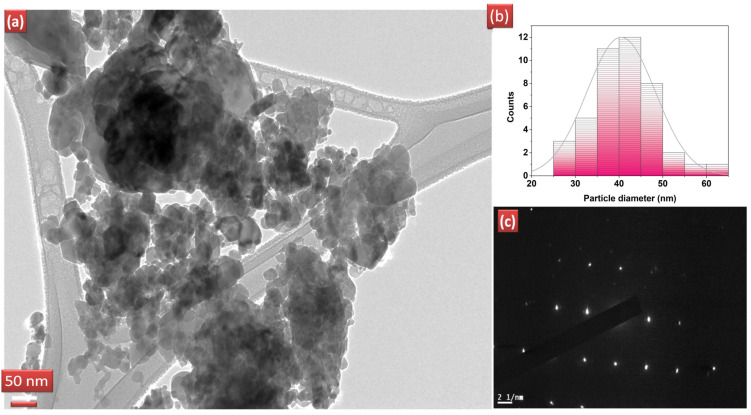
(**a**) TEM image for Dy doped ZnO nanoparticles, (**b**) particle size distribution, (**c**) corresponding SAED pattern.

The surface morphology of the catalysts was analysed with the help of SEM images. Figure 3 shows the SEM images for pure ZnO and REdoped ZnO nanoparticles recorded at the 500 nm scale. It is clear from the images that grains in all samples lie in the nanometer range. The surface of all samples contains almost spherical, rough grains. The surface of pure ZnO appears to have fluffier grains, while the surface of samples doped with rare earth elements looks quite dense. The Nd-doped ZnO nanoparticles appear to have a denser structure than other catalysts. The grains distribution was studied by analyzing the histogram prepared using ImageJ software. For pure ZnO nanoparticles, the grains were found to be in the 10–35 nm range. However, for Sm doped ZnO nanoparticles, grain size distribution ranges between 10–40 nm, with an average grain size of 23 nm. The Dy doped samples consist of grains spreading over a wide range of 5–40 nm, with an average grain size of 22.5 nm. However, for Nd doped ZnO nanoparticles, grains were found to be in the narrow range of 22–40 nm, with an average grain size of 28 nm. The observed grains size for all samples differs slightly from the crystallite size obtained from XRD due to the smaller dimension and agglomerated nature of the samples. The presence and distribution of elements on the surface of the synthesized samples were investigated using energy-dispersive X-ray (EDX) spectroscopy. Figure 4a–d shows the EDX pattern for all synthesized samples. The EDX spectrum revealed the presence of all desired elements in all samples. Furthermore, a TEM image for Dy doped ZnO nanoparticles was recorded (Figure 5a). It is visible from the micrograph that the sample consists of particles smaller than 100 nm. The particle size distribution graph revealed an average particle size of 40 nm for Dy doped ZnO nanoparticles (Figure 5b). The SAED pattern (Figure 5c) consists of bright, regular spots, indicating the good crystalline nature of the sample. The observed results are in good agreement with the XRD results.

### 2.3. Tailoring of the Band Gap

**Figure 6:**
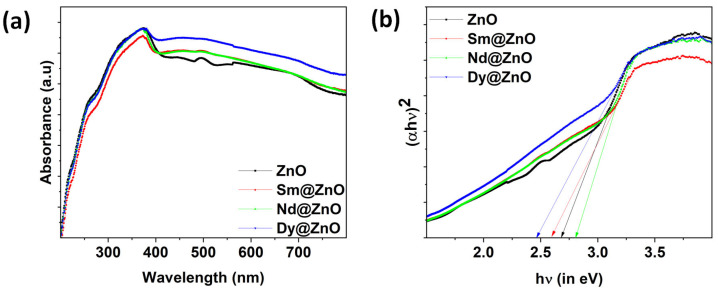
(**a**) Absorbance spectra for all synthesized samples, (**b**) Tauc’s plot obtained using absorbance data.

A UV-vis analysis was employed to investigate the effect of doping ions on ZnO NPs. Figure 6a demonstrates the UV–visible spectra of ZnO, 3% Sm-ZnO, 3% Nd-ZnO, and 3% Dy-ZnO nanoparticles. The absorption edges lie at 382 nm in pure ZnO, mainly because of the electronic transition from the valence band to the conduction band of ZnO nanoparticles [31,32]. It is visible from the absorbance spectra that all samples are capable of absorbing in the visible light region. The rare earth ion doping causes a shift in the absorption edge. As per the literature, the shift in the absorption edge can be related to the charge transfer transition between the f-block electrons of rare earth metal ions and the VB or CB of ZnO nanoparticles [33]. Plotting the Tauc’s curve from absorbance data allows for the precise analysis of band gap variation. The band gap energy value of ZnO and rare earth doped ZnO nanoparticles was calculated using Tauc’s equation given by: ref. [34].

(7) $${\left(\alpha h\nu \right)}^{2}=A\left(h\nu -E_{g}\right)$$

where *α* corresponds to the absorption coefficient, *hν* is known as photon energy, A indicates the band tailing parameter, and *E_g_* denotes the optical band gap. Figure 6b depicts the Tauc’s plot as plotted using Equation (1). The band gap for ZnO, Sm doped, Nd doped, and Dy doped samples were found to be 2.68 eV, 2.59 eV, 2.80 eV, and 2.47 eV, respectively. However, the apparent low band gap can be attributed to the presence of excess Zn, or Zn interstitials in the wurtzite structure of ZnO. In a recent report, band gap values were found to vary from 3.185 to 2.892 eV for un-doped ZnO and 5% Co doped ZnO nanoparticles [35]. Similar results have been reported by P. Pascariu et al. for rare earth doped ZnO nanostructures in which the band gap was 2.80, 2.81 eV, 2.82 eV, and 2.85 eV for un-doped ZnO, Sm, Er and La doped ZnO, respectively [36]. There are claims that even the co-doping (Fe+Al) may lead to a decrease in the band gap of ZnO nanoparticles synthesized through the co-precipitation method, and may improve the optoelectronic properties of ZnO nanoparticles [37]. The band gap energy of a semiconductor material is generally determined by the crystal imperfection caused by the dopant type and amount, as well as other factors. Crystallite size is one of the common factors which generally affects the band gap values; however, in our experimental findings, this does not appear to be the prime factor. The second factor that may have altered the band gap is the presence of some structural defects which are different for un-doped and doped ZnO nanoparticles [38]. The results indicate that rare earth ions are capable of showing a wide absorbance spectrum in sunlight, which can favor the photocatalytic performance of the catalysts.

### 2.4. Elemental Composition

**Figure 7:**
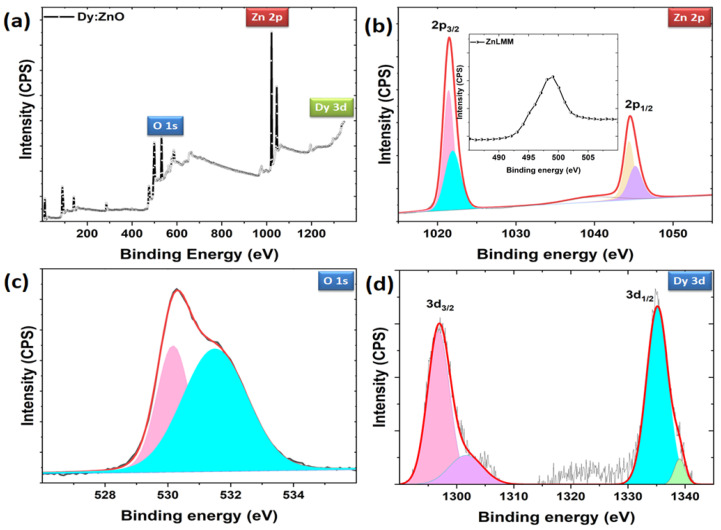
(**a**) Survey scan XPS spectrum for Dy doped ZnO nanoparticles and de-convoluted core level spectra for (**b**) Zn 2p, (**c**) O 1s, and (**d**) Dy 3d level.

To determine the elemental composition of the prepared nanoparticles, the X-ray photoelectron spectra were recorded. The valence state information is analyzed by taking the core level spectra of Zn2p, O1s, and Dy3d. Figure 7a shows the survey scan spectrum for Dy-doped ZnO nanoparticles, confirming the presence of Zn, O, and Dy elements in the sample. The Zn2p spectra contain two major doublets (2p_3/2_, 2p_1/2_), which are further fitted with two major and two minor peaks (Figure 7b). The peaks are located at 1021.41, 1021.94, 1044.40, and 1045.19 eV, respectively. The peaks revealed that Zn ions have a 2+ valence state [39]. No Zn clusters were supported through XPS peaks. The sharpness of the doublet indicates the presence of Zn^2+^ ions in the sample [40]. Two minor peaks correspond to the different environments of Zn ions, indicating Zn interstitials or any defects present. The Zn interstitials can be confirmed through Zn LMM spectra for auger electrons. The Zn LMM auger peak (at 498 eV) from the spectra (as shown inset) is attributed to Zn interstitials [41]. However, to confirm the presence of Zn vacancies in the sample, photoluminescence studies are shown in a later section. The O1s XPS spectrum appeared as an asymmetric curve, which indicates the presence of different oxygen environments in the sample (Figure 7c). The O 1s spectrum is de-convoluted into two peaks at 530.16 and 531.5 eV, respectively. The low binding energy peak is the characteristic peak belonging to Zn-O bonding [42], and the higher binding energy peak is an indication of loosely bound oxygen or adsorbed oxygen on the surface of the sample [43]. The Dy core level spectra consists of two major peaks at 1296.98 and 1335.36 eV, which confirms the Dy ions in the 3+ state in the sample (Figure 7d) [24,44]. Overall, the XPS results confirm the presence of OH groups on the surface of the catalyst and the valence states of 2+ and 3+ for Zn and Dy ions.

### 2.5. Defect Analysis

**Figure 8:**
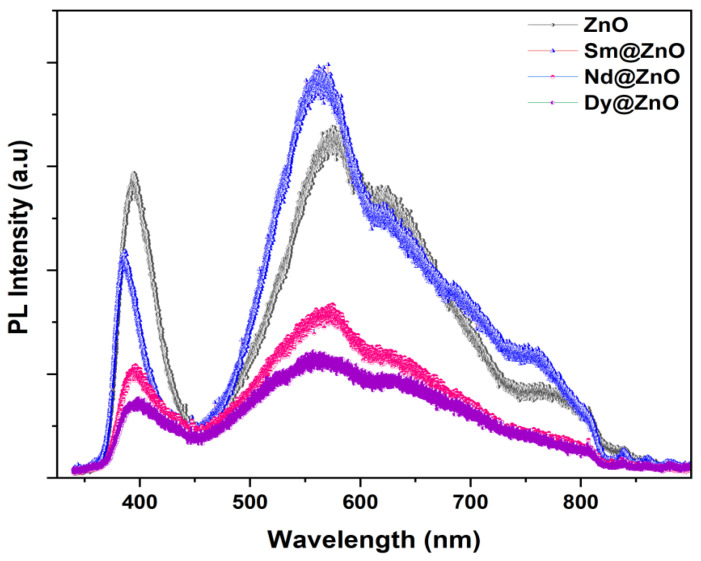
Room temperature PL spectra for all synthesized samples.

The reason for the variation in the photocatalytic performance of the catalysts can be understood by analyzing the photoluminescence emission spectra of all samples. Figure 8 represents the PL spectra for pure ZnO and doped samples. The charge separation efficiency of the carriers is verified by analyzing the PL emission efficiency in the observed spectra. At an excitation wavelength of 320 nm, the emission spectra of pure ZnO nanoparticles and rare earth doped nanoparticles were recorded at room temperature. The lower PL intensity symbolizes a lower charge recombination rate, while the higher PL intensity shows a higher charge recombination rate of the charge carriers. In the UV region, pure ZnO exhibits the highest PL intensity, followed by the Sm, Nd and Dy doped ZnO samples. However, in the visible region, PL intensity follows the trend of Sm:ZnO > ZnO > Nd:ZnO > Dy:ZnO. The results indicate that the lowest electron-hole recombination is favoured for Dy doped ZnO nanoparticles as compared to other photocatalysts, thereby altering their photocatalytic performance in the degradation of pollutants [45].

**Figure 9:**
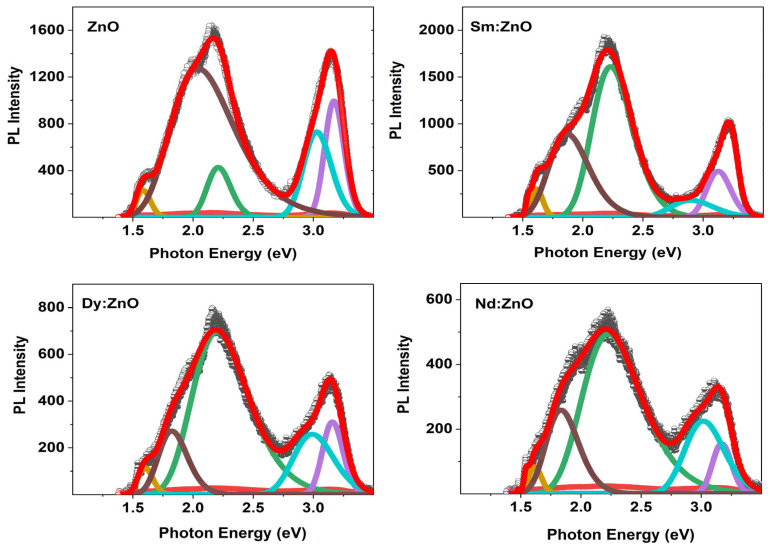
Gaussian fitted PL spectra for all synthesized samples.

For further information, the PL spectra of all samples are Gaussian fitted (Figure 9). According to reports, a PL spectrum of ZnO shows two bands, one in the UV region and the other in the visible region. The first region is assigned to the near-band-edge emission between exciton-exciton collision processes. The second one is due to electron-hole recombination originating from the presence of intrinsic point defects and surface defects like oxygen vacancies, zinc interstitials, and adsorbed hydroxyl groups in the crystal lattice in the synthesis process. The defect related band is de-convoluted into three bands. As per reports, ~480–550 nm band (blue-green emission), ~550–610 nm (yellow-emission), while ~610–750 nm (orange-red emission) indicate the presence of single charged oxygen vacancy (V_0_^+^), doubly charged oxygen vacancy (V_0_^++^) and excess oxygen [46]. Moreover, the violet-blue emission band (410–470 nm) signifies the presence of Zn interstitials in the ZnO structure [47]. Table 2 lists all observed PL peaks and their origin. The PL results show the presence of Zn vacancies, O vacancies, Zn interstitials, and excess oxygen in the catalysts. These results are well supported by the observed XPS results.

### 2.6. Magnetic Properties

**Figure 10:**
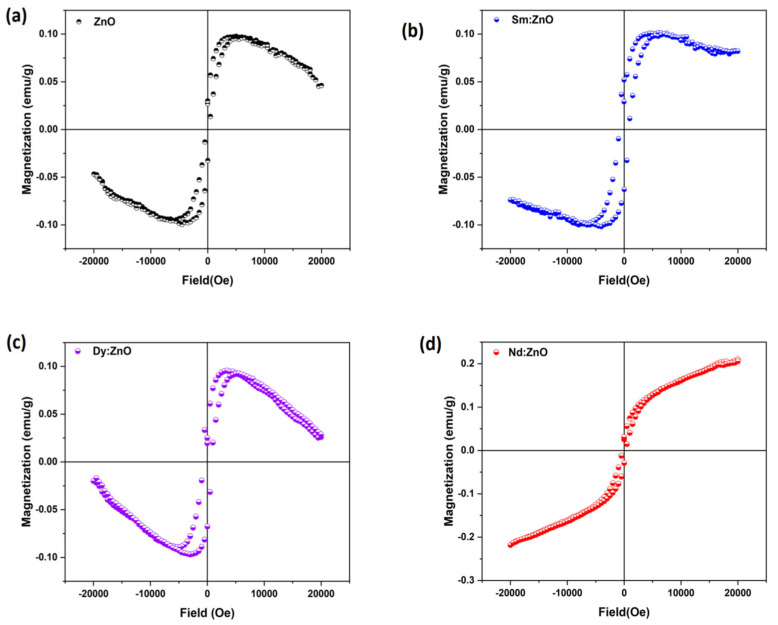
Room temperature magnetization vs. magnetic field (M-H loops for (**a**) ZnO (**b**) Sm:ZnO (**c**) Dy:ZnO (**d**) Nd:ZnO.

The magnetization versus applied magnetic field curves recorded at room temperature for all samples are shown in Figure 10(a–d). M-H loops of bare ZnO nanoparticles (Figure 10a), as well as Sm and Dy doped ZnO nanoparticles (Figure 10b,c), are divided into two types of regions. One is for low fields around the origin of the curves, showing the magnetic nature of the samples, and the other is for high field values, confirming the diamagnetic contribution in the samples as well.

The observed magnetic behaviour in synthesized nanoparticles of bare ZnO can be attributed to the presence of defects in the materials, as ZnO in general belong to the diamagnetic materials family. Other researchers have reported the presence of both ferromagnetism and diamagnetism in ZnO nanoparticles doped with rare earth elements [48]. For Sm doped ZnO nanoparticles, the diamagnetic contribution appeared to be weak, while ferromagnetic interactions dominated, resulting in a well-defined hysteresis loop. In the case of Dy doped ZnO nanoparticles (Figure 10d), diamagnetic and ferromagnetic interactions both exist at the same time. Such a shape of the hysteresis curve arises from competing ferromagnetic and diamagnetic interactions. The observed room temperature hysteresis loop for Nd doped ZnO nanoparticles is different from that for pure ZnO and Sm, Dy doped ZnO nanoparticles. The Nd doping results in magnetic behavior; however, the magnetization does not saturate at higher strengths of the applied magnetic field. Such behaviour is typical of nanoparticles where ferromagnetic and antiferromagnetic interactions compete with each other [49]. There is still much disagreement over the cause of the RTFM effect seen in metal oxide compounds, and it may be highly influenced by processing variables such as the synthesis technique, temperature, grain size, annealing treatment, and most likely doping or co-doping [50]. The observed magnetism in our synthesized samples can be attributed to the presence of defects, as confirmed from PL studies. As no secondary phase was confirmed by XRD studies, the cause of magnetism may not be considered extrinsic. The observed magnetism may be considered intrinsic magnetism and could be utilized in spintronic devices.

## 3. Photodegradation Experiment

The fabricated photocatalysts were employed for the degradation of tetracycline drugs and even for pollutant dyes like malachite green (MG) and crystal violet (CV) under the irradiation of UV-visible light (a Xenon lamp).

### Degradation of Tetracycline

**Figure 11:**
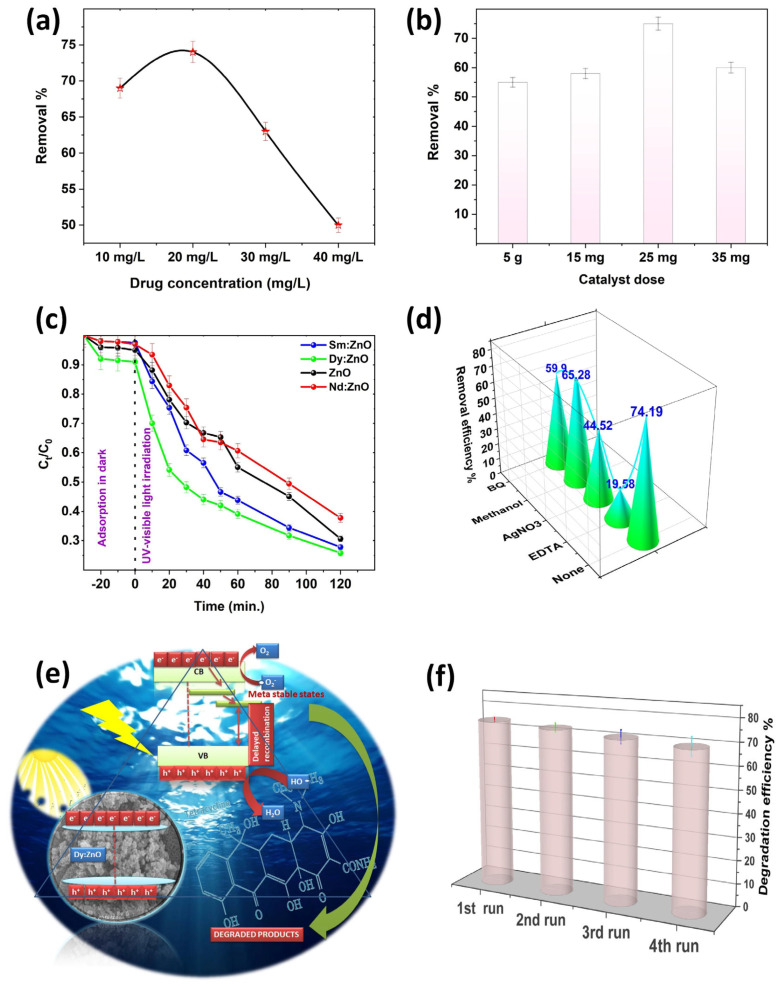
(**a**) Effect of TC concentration on photodegradation efficiency, (**b**) Effect of catalyst dosage on photodegradation efficiency of Dy doped ZnO nanoparticles, (**c**) C_t_/C_0_ graph for degradation of TC in a 120 min experiment (reaction conditions: catalyst dosage: 25 mg, drug concentration: 20 mg/L, temperature: 20 ± 5 °C), (**d**) Calculated removal efficiency in the presence of various scavengers, (**e**) Schematic of plausible mechanism for the degradation of TC, (**f**) Reusability and sustainability analysis of Dy doped ZnO catalyst.

In the experiment, the TC concentration and catalyst dosage were optimized by performing the degradation experiments for varied TC concentrations (10, 20, 30, 40 mg/L) and catalyst (Dy doped ZnO) dosages (5, 15, 25, 35 mg). The observed results are shown in Figure 11a,b. The initial concentration of organic matter is a crucial factor to take into account when investigating photocatalytic reactions, since it directly influences the rate and level of degradation of the entire catalytic reaction. When the catalyst dose was 25 mg, the maximum TC degradation was observed at 20 mg/L TC concentration. Therefore, 20 mg/L TC concentration was considered as the optimum concentration for a photocatalytic degradation experiment. In addition, the dosage of the catalyst is also as crucial factor for the photocatalytic reactions, and the amount of catalyst was optimized at a TC concentration of 20 mg/L, as shown in Figure 11b. The chart clearly shows that the 25 mg of catalyst has the maximum degrading efficiency. The degradation rate reduces when the amount of catalyst becomes too high, which might be owing to the fact that an optimal amount of catalyst can increase the number of reactants and catalyst particles, resulting in enhanced catalytic efficiency. When the catalyst concentration is too high, the presence of surplus particles causes light dispersion and blocking of the light route, resulting in a lower chance of the surface of the catalyst particles receiving uv-visible light and, as a result, a drop in reaction efficiency. Finally, the photocatalytic degradation performance is optimum, with an initial TC concentration of 20 mg/L and a catalyst dose of 25 mg.

**Figure 12:**
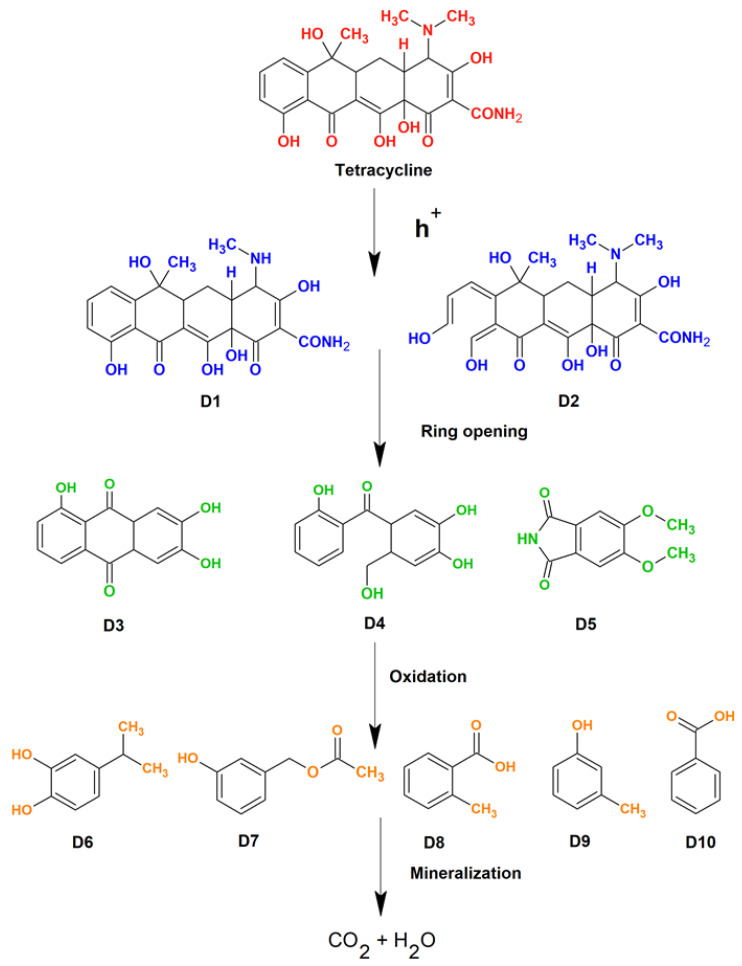
Schematics showing reaction intermediates in the detailed degradation pathway.

In a further experiment, the system was initially equilibrated by mixing the catalyst (25 mg) with target pollutant solutions for 30 min in the dark. The initial antibiotic concentration was fixed at 20 mg/L. Following this, the light source was turned on, and the TC concentration degradation was monitored as the photocatalysis process continued. Figure 11c shows the observed C_t_/C_0_ curves for the degradation of tetracycline (TC) in the presence of different synthesized catalysts. According to the results, the introduction of rare-earth elements into the ZnO matrix increased the photocatalytic capabilities of the Dy and Sm doped materials, while Nd doped ZnO samples showed a slight decrease when compared to ZnO nanoparticles. The results indicate that Dy doping on Zn sites has resulted in increased degradation efficiency. The catalytic efficacy is determined by the rates of the redox reactions involved in the degradation process. It is worth noting that the deterioration is much quicker in the first few minutes of light irradiation, which is due to the faster production of reactive species responsible for degradation at the beginning of the experiment. The rate of the degradation of the pollutant relies on several factors such as irradiation time, light source type, intensity, catalyst dosage, target pollutant concentration, temperature conditions, pH, etc. [51] All of these factors play crucial roles in determining the photodegradation efficiency of the catalysts. For more clarity, the rate constant of the reactions were determined using a pseudo first order kinetics equation. The observed rate constant follows the following order: Dy doped ZnO (0.0101 min^−1^) > ZnO (0.009410 min^−1^) > Sm doped ZnO (0.009069 min^−1^) > Nd doped ZnO (0.007863 min^−1^) nanoparticles. Scavenging experiments on Dy doped ZnO photocatalysts were carried out to confirm the photodegradation mechanism involved in the degradation of TC. The degradation experiments were performed with or without the presence of scavenging agents, namely benzoquinone (BQ), methanol, AgNO_3_, and ethylene diamine tetraacetic acid (EDTA) for superoxide radical, hydroxyl radicals, electrons, and holes, respectively. Figure 11d presents the degradation efficiency achieved in the presence of different scavengers. The calculated degradation efficiency without any scavengers was found to be 74.19%. With the addition of BQ and methanol, the removal efficiency of the Dy-doped ZnO catalyst decreased slightly, indicating that the superoxide and hydroxyl radicals are not directly involved in the degradation process. However, the role of holes as reactive species in redox reactions was found to be evident, as revealed by the scavenging of removal efficiency on EDTA addition. The addition of EDTA significantly reduced the removal efficiency of the Dy doped catalyst to 19.68%. The results indicate that the positively charged holes directly participate in the oxidation of H_2_O to form ^•^OH radicals, while photogenerated electrons convert the dissolved oxygen to produce superoxide radicals. The schematics of the involved mechanisms are shown in Figure 11e. The results are in favour of holes as primary reactive species, which helps in the mineralization of TC. As semiconductor nanomaterials are exposed to light with energy greater than or equal to the band gap, an electron in the valence band can be stimulated to move to the conduction band, resulting in the formation of a hole in the VB. The photoelectron is easily caught by electronic acceptors such as adsorbed O_2_ to generate a superoxide radical anion, but the photo induced holes are easily trapped by electron donors to oxidise organic contaminants [52]. In the case of Dy doped ZnO nanoparticles, the presence of adsorbed oxygen is supported by XPS results. Aside from interacting with electron donors or acceptors adsorbed on the semiconductor surface, photoinduced electrons and holes can either recombine or exhaust the input energy as heat or get stranded in metastable states. However, the recombination is inhibited if a sufficient scavenger or defect state is present to capture the electron or hole, and further redox reactions can proceed. PL analysis along with XPS interpretation confirmed the presence of defect states and meta-stable states in the rare earth doped ZnO nanoparticles, resulting in delayed recombination and better charge separation in the synthesized photo catalysts. To check the reusability and sustainability of the Dy doped ZnO nanoparticles as catalysts, the degradation experiments were performed for four consecutive cycles (Figure 11f). The results indicated that there is no evident loss in degradation efficiency, even after the fourth cycle of the experiment. To understand the depth of the detailed mechanism and the pathway involved in the process of mineralization, an LC-MS analysis was performed. The detailed pathway is shown in Figure 12. In the first step, the TC molecule undergoes de-methylation via h^+^ oxidation. The oxidation of h^+^ decreases the electron density around the benzene ring, followed by the partial opening of the benzene ring. In the second step, the breakdown of TC takes place by the attack of reactive species on C-C, C-OH, and C-N bonds [53]. Additionally, the formation of short chain structures takes place through the oxidation of the reactive species. In the end, a number of tiny molecular compounds were formed, which were eventually changed into CO_2_ and H_2_O in the carbonization process, accompanied by the occurrence of a ring-opening reaction under the further oxidation of h^+^.

**Figure 13:**
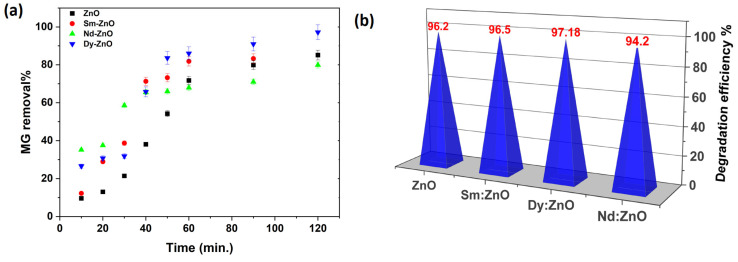
Degradation efficiency against (**a**) MG dye achieved in a 120 min experiment; (**b**) Crystal violet dye.

To evaluate the effectiveness of the produced catalysts, we performed a photocatalytic test on the organic dyes Malachite green (MG) and Crystal violet (CV) under UV-visible light irradiation. The produced materials’ photocatalytic performance was examined by measuring the change in MG concentration as a function of irradiation time in the presence of various catalysts. Dy-doped ZnO was shown to have better photo catalytic activity than the other doped catalysts and pure ZnO for this dye, as shown in Figure 13a. The maximum degradation efficiency of 97.13% was achieved for the Dy doped ZnO photo catalyst, followed by the Sm doped catalyst (96%), ZnO (84%), and the Nd doped catalyst (79%). Another set of experiments was performed for the degradation of crystal violet dye (CV). The results are in accordance with the trend obtained for MG and TC degradation. However, the maximum degradation efficiency in this case was 96.18% for the Dy doped ZnO catalyst. All synthesized catalysts were found to exhibit excellent degradation efficiency (>94%) against CV dye (Figure 13b). The results shows that rare earth doped ZnO nano photocatalysts are very promising for the photodegradation of drugs and dyes. Furthermore, the organic pollutant degradation efficiency of the produced photocatalyst was compared to the prior results, which are shown in Table 3.

## 4. Materials and Methods

### 4.1. Materials

The chemicals used for the synthesis of nanoparticles are hexahydrated zinc nitrate (Merck; 99%), hexahydrated samarium nitrate (Merck; >99%), hexahydrated neodymium nitrate (Merck; >99%), dysprosium nitrate (Merck; 99.9), citric acid (Merck; 99.5%), and ammonia solution (Loba chemicals). Malachite green, Crystal violet, and tetracycline were selected as the model organic pollutants. Triple-distilled water was used for the synthesis of ZnO and the rare earth-doped ZnO nanoparticles.

### 4.2. Methodology

Zinc nitrate and rare earth metal nitrate were used as the appropriate precursors in a sol-gel process to fabricate the various RE (Sm, Nd, and Dy)-doped ZnO nanoparticles. For the synthesis of undoped ZnO nanoparticles, 11.89 g of zinc nitrate was dissolved in 25 mL of distilled water with the use of a magnetic stirrer. The citric acid was also dissolved separately in 25 mL distilled water using the same method. Both of the solutions were mixed after a half hour of continuous stirring. After the above solution was completely dissolved, the ammonia solution was added drop by drop to keep the pH at 7. Then, while continuously stirring, the entire mixture was heated to 80 °C. After the gel formed, it was transferred to a crucible and heated for 4 h at 550 °C in a muffle furnace. Upon cooling, the final product was grounded and stored for further characterization. For the synthesis of Sm doped ZnO nanoparticles, 11.54 g, 0.53 g, and 15.36 g of Zn nitrate, Sm nitrate and citric acid were used as raw materials, respectively. For the Nd doped sample, 11.54 g, 0.52 g, and 15.36 g of Zn nitrate, Nd nitrate and citric acid were utilized, respectively. The same route was followed for the synthesis of Dy doped ZnO nanoparticles using 11.54 g, 0.41 g, and 15.36 g of Zn nitrate, Nd nitrate and citric acid, respectively.

X-ray diffraction (X’Pert Pro, Malvern Panalytical, Malvern, UK) was used to determine the crystal structure as well as other structural parameters of the fabricated photo catalysts. Scanning electron microscope (SEM; SU8010, Hitachi High-Technologies, Tokyo, Japan) micrographs were used to examine the surface morphology of the produced catalysts. The particle size analysis was performed using a transmission electron microscope (TEM H-7500, Hitachi High-Technologies, Tokyo, Japan). The valence state determination of the elements was performed using an X-ray photoelectron spectrometer (XPS; Nexsa base, Thermofisher Scientific, Waltham, MA, USA). The bandgap was identified by tracking the absorbance spectra using a UV-VIS-NIR spectrophotometer (1900i, Shimadzu, Japan). We collected data on the magnetic properties of the materials by measuring their M-H curves with a vibrating sample magnetometer (EV7, MicroSense, Lowell, MA, USA). A fluorescence spectrometer was utilised to determine the photoluminescence spectra for the produced photocatalysts (F-4600, 325 nm excitation wavelength, Hitachi High-Technologies, Tokyo, Japan).

### 4.3. Photodegradation Experiment

The synthesized photocatalysts were utilized for the degradation of tetracycline (TC) at room temperature under UV-visible light irradiation (Xe lamp, 500 W) in a photochemical reactor fitted with a magnetic stirrer and a water circulating arrangement to avoid unwanted heating. The experiment began with 25 mg of catalyst added to 100 mL of a 20 mg/L TC solution. The mixture was then placed under a xenon lamp. At regular intervals, equilots from the reaction suspension were injected out with a syringe. The membrane filter was then used to get rid of any unwanted catalyst particles. Using an HPLC equipped with a C18 column and a UV detector at 288 nm, the concentration of residual TC was determined. As a mobile phase, 15% methanol and 1% formic acid in ultrapure water (85%) were utilized. In the positive electrospray ionization (ESI) mode, TC degradation intermediates were studied and identified by LC-MS. The percentage degradation of TC was calculated using the following formula [60]:

(8) $$\%\;degradation=\frac{C_{0-}C_{t}}{C_{0}}X100$$

The degradation kinetics was analyzed with the pseudo first order kinetic equation given by [61]:

(9) $$ln\frac{C_{0}}{C_{t}}=k_{app}t$$

A similar procedure was chosen for the Malachite green (MG) and Crystal violet (CV) dye degradation experiment.

## 5. Conclusions

Highly crystalline pure phase undoped ZnO and rare earth doped ZnO nanoparticles were synthesized through an easy, fast and economic route for analyzing their photodegradation efficiency for the removal of tetracycline, Malachite green, and Crystal violet dye. The opted synthesis route and growth conditions resulted in the emergence of a large number of lattice defects (Zn_in_/V_Zn_, V_o_, excess oxygen etc.) in undoped and doped ZnO nanoparticles. Among ZnO, Sm, Nd, Dy doped ZnO nano-catalysts, the Dy doped ZnO catalyst exhibited maximum degradation efficiency (74.19%; TC, 97.18; MG, 98%; CV) against the targeted pollutants. In addition, the catalyst shows a sustainable nature, and can be reused after four cycles, as confirmed from reusability experiments. The trapping experiments support the holes as highly reactive species in the degradation of TC. The elevated degradation efficiency of the catalyst has been explained in view of a tailored band gap and the presence of defects, which helped in the creation of a reactive species responsible for the removal of organic pollutants. The PL and XPS analysis demonstrated the presence of the lattice defects. The overall results indicate that the fabricated catalysts can be successfully employed for wastewater treatment for the degradation of resistant drugs such as TC and other pollutants. Moreover, the obtained magnetic properties suggest the application of synthesized catalysts in spintronic applications as well.

## Figures and Tables

**Table 1:** Structural parameters of rare earth doped ZnO nanoparticles.

Structural Parameters		ZnO	Sm-ZnO	Nd-ZnO	Dy-ZnO
Crystal Size (nm)		19.17	12.04	13.46	13.12
Lattice Parameters	a = b (Å)	3.242	3.237	3.235	3.241
	c (Å)	5.201	5.198	5.195	5.003
Strain		0.00357	0.00356	0.00357	0.00356
V (Å)^3^		47.33	47.13	47.05	45.49
Bond-Length (Zn-O) (Å)		1.974	1.971	1.969	1.945
R		1.017	1.016	1.016	1.057

**Table 2:** The peak positions and their origin of de-convoluted PL spectra for all samples.

Sample ID	Peak Position (nm)	Photon Energy (eV)	Origin of Emission
ZnO	389.32	3.18	Band edge emission
	409.76	3.02	Zn_in_
	561.89	2.20	V_0_^++^
	604.55	2.05	V_0_^++^
	788.24	1.57	excess oxygen
Sm:ZnO	393.32	3.15	Band edge emission
	432.28	2.86	Zn_in_
	558.48	2.22	V_0_^++^
	662.47	1.87	excess oxygen
	771.94	1.60	excess oxygen
Nd:ZnO	391.10	3.17	Band edge emission
	415.24	2.98	V_Zn_
	565.59	2.19	V_0_^++^
	682.47	1.81	excess oxygen
	777.13	1.59	excess oxygen
Dy:ZnO	391.54	3.16	Band edge emission
	413.76	2.99	V_Zn_
	562.19	2.20	V_0_^++^
	679.06	1.82	excess oxygen
	773.72	1.60	excess oxygen

**Table 3:** Comparison of previously reported work on various pollutants’ degradation using ZnO-based photo catalysts.

Sr. No.	Photocatalyst	Pollutant	Light Source	Drug Concentration	Catalyst Dosage	Removal Efficiency (%) (Reaction Time)	Reference
1	Gd/La@ZnO	Tetracycline	Sunlight	10 mg/L	100 mg	74%, (100 min)	[54]
2	1.5% Gd-Nd doped ZnO	Methlene blue	300 W tungsten lamp	20 mg/L	100 mg	93% (120 min)	[55]
3	0.2% La-ZnO-SiO_2_	Malachite green	300 W xenon lamp	15 mg/L	15 mg	96% (140 min)	[56]
4	SDS/ZnO	Tetracycline	350 W xenon lamp	40 mg/L	20 mg	49% (150 min)	[16]
5	PVDF/ZnO:Ln fibrous mat	Methlene blue, Rhodamine B	400 W xenon lamp	10 mg/L, 5 mg/L	1 g	96.33%, 93.36% (360 min)	[57]
6	ZnO single bond SnO_2_ (70%)	Tetracycline	300 W xenon lamp	1 g/L	60 mg	70.9% (50 min)	[58]
7	Ag/ZnO@BC	Tetracycline	500 W xenon lamp	50 mg/L	10 mg	70.3% (60 min)	[59]
8	Dy doped ZnO	Tetracycline, Malachite Green, Crystal Violet	500 W xenon lamp	20 mg/L	25 mg	74.90%, 97.18%, 98% (120 min)	This work

## Data Availability

Data is contained within the article.

## Supplementary Materials

The following supporting information can be downloaded at: https://www.mdpi.com/article/10.3390/molecules28062838/s1, Raw data for W-H@R-ZNO plots.

## Sample Availability

Samples of the compounds are not available from the authors.

## References

[1] Ahmad F., Zhu D., Sun J. (2021). Environmental fate of tetracycline antibiotics: Degradation pathway mechanisms, challenges, and perspectives. Environ. Sci. Eur..

[2] Saadati F., Keramati N., Ghazi M.M. (2016). Influence of parameters on the photocatalytic degradation of tetracycline in wastewater: A review. Crit. Rev. Environ. Sci. Technol..

[3] Khan I., Saeed K., Zekker I., Zhang B., Hendi A.H., Ahmad A., Ahmad S., Zada N., Ahmad H., Shah L.A. (2022). Review on Methylene Blue: Its Properties, Uses, Toxicity and Photodegradation. Water.

[4] Mancuso A., Blangetti N., Sacco O., Freyria F.S., Bonelli B., Esposito S., Sannino D., Vaiano V. (2023). Photocatalytic Degradation of Crystal Violet Dye under Visible Light by Fe-Doped TiO_2_ Prepared by Reverse-Micelle Sol&ndash;Gel Method. Nanomaterials.

[5] Gul K., Khan H., Muhammad N., Ara B., Zia T.U.H. (2021). Removal of toxic malachite green dye from aqueous environment using reduced magnetic graphene oxide as an efficient and reusable adsorbent. Sep. Sci. Technol..

[6] Skjolding L.M., Jørgensen L.V., Dyhr K.S., Köppl C.J., McKnight U.S., Bauer-Gottwein P., Mayer P., Bjerg P.L., Baun A. (2021). Assessing the aquatic toxicity and environmental safety of tracer compounds Rhodamine B and Rhodamine WT. Water Res..

[7] Saeed M., Muneer M., Haq A.U., Akram N. (2022). Photocatalysis: An effective tool for photodegradation of dyes—A review. Environ. Sci. Pollut. Res..

[8] Chen H., Goswami D., Stefanakos E. (2010). Renewable and Sustain. Energy Rev..

[9] Dhiman P., Rana G., Kumar A., Sharma G., Vo D.-V.N., Naushad M. (2022). ZnO-based heterostructures as photocatalysts for hydrogen generation and depollution: A review. Environ. Chem. Lett..

[10] Ahmad S., Almehmadi M., Janjuhah H.T., Kontakiotis G., Abdulaziz O., Saeed K., Ahmad H., Allahyani M., Aljuaid A., Alsaiari A.A. (2023). The Effect of Mineral Ions Present in Tap Water on Photodegradation of Organic Pollutants: Future Perspectives. Water.

[11] Hemalatha P., Karthick S., Hemalatha K., Yi M., Kim H.-J., Alagar M. (2016). La-doped ZnO nanoflower as photocatalyst for methylene blue dye degradation under UV irradiation. J. Mater. Sci. Mater. Electron..

[12] Chang X., Li Z., Zhai X., Sun S., Gu D., Dong L., Yin Y., Zhu Y. (2016). Efficient synthesis of sunlight-driven ZnO-based heterogeneous photocatalysts. Mater. Des..

[13] Dhiman P., Sharma S., Kumar A., Shekh M., Sharma G., Naushad M. (2020). Rapid visible and solar photocatalytic Cr(VI) reduction and electrochemical sensing of dopamine using solution combustion synthesized ZnO–Fe_2_O_3_ nano heterojunctions: Mechanism Elucidation. Ceram. Int..

[14] Wang Y., Zhao X., Duan L., Wang F., Niu H., Guo W., Ali A. (2015). Structure, luminescence and photocatalytic activity of Mg-doped ZnO nanoparticles prepared by auto combustion method. Mater. Sci. Semicond. Process..

[15] Dhiman P., Chand J., Kumar A., Kotnala R.K., Batoo K.M., Singh M. (2013). Synthesis and characterization of novel Fe@ZnO nanosystem. J. Alloys Compd..

[16] Jia K., Liu G., Lang D.-N., Chen S.-F., Yang C., Wu R.-L., Wang W., Wang J.-D. (2022). Degradation of tetracycline by visible light over ZnO nanophotocatalyst. J. Taiwan Inst. Chem. Eng..

[17] Shkir M., Palanivel B., Khan A., Kumar M., Chang J.-H., Mani A., AlFaify S. (2022). Enhanced photocatalytic activities of facile auto-combustion synthesized ZnO nanoparticles for wastewater treatment: An impact of Ni doping. Chemosphere.

[18] Khatamian M., Khandar A., Divband B., Haghighi M., Ebrahimiasl S. (2012). Heterogeneous photocatalytic degradation of 4-nitrophenol in aqueous suspension by Ln (La3+, Nd3+ or Sm3+) doped ZnO nanoparticles. J. Mol. Catal. A Chem..

[19] Selvam N.C.S., Vijaya J.J., Kennedy L.J. (2013). Comparative studies on influence of morphology and La doping on structural, optical, and photocatalytic properties of zinc oxide nanostructures. J. Colloid Interface Sci..

[20] Labhane P.K., Sonawane G.H., Sonawane S.H. (2018). Influence of rare-earth metal on the zinc oxide nanostructures: Application in the photocatalytic degradation of methylene blue and p-nitro phenol. Green Process. Synth..

[21] Vaiano V., Matarangolo M., Sacco O., Sannino D. (2017). Photocatalytic treatment of aqueous solutions at high dye concentration using praseodymium-doped ZnO catalysts. Appl. Catal. B Environ..

[22] Zong Y., Li Z., Wang X., Ma J., Men Y. (2014). Synthesis and high photocatalytic activity of Eu-doped ZnO nanoparticles. Ceram. Int..

[23] Korake P., Kadam A., Garadkar K. (2014). Photocatalytic activity of Eu^3+^-doped ZnO nanorods synthesized via microwave assisted technique. J. Rare Earths.

[24] Ökte A.N. (2014). Characterization and photocatalytic activity of Ln (La, Eu, Gd, Dy and Ho) loaded ZnO nanocatalysts. Appl. Catal. A Gen..

[25] Nguyen T.H.A., Le V.T., Doan V.-D., Tran A.V., Nguyen V.C., Nguyen A.-T., Vasseghian Y. (2022). Green synthesis of Nb-doped ZnO nanocomposite for photocatalytic degradation of tetracycline antibiotic under visible light. Mater. Lett..

[26] Singh S., Kumar V., Tyagi S., Saxena N., Khan Z.H., Kumar P. (2022). Room temperature ferromagnetism in metal oxides for spintronics: A comprehensive review. Opt. Quantum Electron..

[27] Zhang X.J., Mi W.B., Wang X.C., Bai H.L. (2014). First-principles prediction of electronic structure and magnetic ordering of rare-earth metals doped ZnO. J. Alloys Compd..

[28] Sin J.-C., Lam S.-M., Lee K.-T., Mohamed A.R. (2013). Preparation and photocatalytic properties of visible light-driven samarium-doped ZnO nanorods. Ceram. Int..

[29] Navarro-López D.E., Garcia-Varela R., Ceballos-Sanchez O., Sanchez-Martinez A., Sanchez-Ante G., Corona-Romero K., Buentello-Montoya D., Elías-Zuñiga A., López-Mena E.R. (2021). Effective antimicrobial activity of ZnO and Yb-doped ZnO nanoparticles against Staphylococcus aureus and Escherichia coli. Mater. Sci. Eng. C.

[30] Dhiman P., Rana G., Kumar A., Sharma G., Vo D.-V.N., AlGarni T.S., Naushad M., ALOthman Z.A. (2021). Nanostructured magnetic inverse spinel Ni–Zn ferrite as environmental friendly visible light driven photo-degradation of levofloxacin. Chem. Eng. Res. Des..

[31] Zhang L.-S., Wong K.-H., Yip H.-Y., Hu C., Yu J.C., Chan C.-Y., Wong P.-K. (2010). Effective photocatalytic disinfection of E. coli K-12 using AgBr−Ag−Bi2WO6 nanojunction system irradiated by visible light: The role of diffusing hydroxyl radicals. Environ. Sci. Technol..

[32] Poornaprakash B., Chalapathi U., Reddy B.P., Vattikuti S.P., Reddy M.S.P., Park S.-H. (2018). Elemental, morphological, structural, optical, and magnetic properties of erbium doped ZnO nanoparticles. Mater. Res. Express.

[33] Zhang L., Yang Y., Fan R., Yu J., Li L. (2013). Improving the efficiency of ZnO-based dye-sensitized solar cells by Pr and N co-doping. J. Mater. Chem. A.

[34] Dhiman P., Naushad M., Batoo K.M., Kumar A., Sharma G., Ghfar A.A., Kumar G., Singh M. (2017). Nano FexZn1−xO as a tuneable and efficient photocatalyst for solar powered degradation of bisphenol A from aqueous environment. J. Clean. Prod..

[35] Saadi H., Benzarti Z., Sanguino P., Pina J., Abdelmoula N., de Melo J.S.S. (2023). Enhancing the electrical conductivity and the dielectric features of ZnO nanoparticles through Co doping effect for energy storage applications. J. Mater. Sci. Mater. Electron..

[36] Pascariu P., Cojocaru C., Olaru N., Samoila P., Airinei A., Ignat M., Sacarescu L., Timpu D. (2019). Novel rare earth (RE-La, Er, Sm) metal doped ZnO photocatalysts for degradation of Congo-Red dye: Synthesis, characterization and kinetic studies. J. Environ. Manag..

[37] Saadi H., Benzarti Z., Sanguino P., Hadouch Y., Mezzane D., Khirouni K., Abdelmoula N., Khemakhem H. (2022). Improving the optical, electrical and dielectric characteristics of ZnO nanoparticles through (Fe + Al) addition for optoelectronic applications. Appl. Phys. A.

[38] Ekennia A.C., Uduagwu D.N., Nwaji N.N., Oje O.O., Emma-Uba C.O., Mgbii S.I., Olowo O.J., Nwanji O.L. (2021). Green synthesis of biogenic zinc oxide nanoflower as dual agent for photodegradation of an organic dye and tyrosinase inhibitor. J. Inorg. Organomet. Polym. Mater..

[39] Dhiman P., Kumar A., Shekh M., Sharma G., Rana G., Vo D.-V.N., AlMasoud N., Naushad M., ALOthman Z.A. (2021). Robust magnetic ZnO-Fe2O3 Z-scheme hetereojunctions with in-built metal-redox for high performance photo-degradation of sulfamethoxazole and electrochemical dopamine detection. Environ. Res..

[40] Morozov I.G., Belousova O.V., Ortega D., Mafina M.K., Kuznetcov M.V. (2015). Structural, optical, XPS and magnetic properties of Zn particles capped by ZnO nanoparticles. J. Alloys Compd..

[41] Yao Z., Tang K., Ye J., Xu Z., Zhu S., Gu S. (2016). Identification and control of native defects in N-doped ZnO microrods. Opt. Mater. Express.

[42] Kalaiezhily R.K., Saravanan G., Asvini V., Vijayan N., Ravichandran K. (2018). Tuning violet to green emission in luminomagnetic Dy,Er co-doped ZnO nanoparticles. Ceram. Int..

[43] Al-Harbi F.F., El Ghoul J.M. (2021). Sol–Gel Synthesis of Dy Co-Doped ZnO:V Nanoparticles for Optoelectronic Applications. Condens. Matter.

[44] Munirathnam K., Rajavaram R., Nagajyothi P.C., Thiyagaraj S., Srinivas M. (2020). Synthesis and optimization of Dy-doped SrZr4(PO4)6 nanophosphors for plant growth light-emitting diodes. Solid State Sci..

[45] Murugadoss G., Salla S., Kumar M.R., Kandhasamy N., Al Garalleh H., Garaleh M., Brindhadevi K., Pugazhendhi A. (2023). Decoration of ZnO surface with tiny sulfide-based nanoparticles for improve photocatalytic degradation efficiency. Environmental Research.

[46] Qi B., Olafsson S., Gíslason H. (2017). Vacancy defect-induced d0 ferromagnetism in undoped ZnO nanostructures: Controversial origin and challenges. Prog. Mater. Sci..

[47] Bandopadhyay K., Mitra J. (2015). Zn interstitials and O vacancies responsible for n-type ZnO: What do the emission spectra reveal?. RSC Adv..

[48] Ayon S.A., Jamal M., Billah M.M., Neaz S. (2022). Augmentation of magnetic properties and antimicrobial activities of band gap modified Ho3+ and Sm3+ doped ZnO nanoparticles: A comparative experimental study. J. Alloys Compd..

[49] Li Q., Zhang Y., Zhang M., Cheng W., Liao B., Ying M. (2023). Structural, electrical and magnetic properties of Gd-doped and (Al, Gd) codoped ZnO films. J. Alloys Compd..

[50] Yahmadi B., Kamoun O., Alhalaili B., Alleg S., Vidu R., Kamoun Turki N. (2020). Physical investigations of (Co, Mn) Co-doped ZnO nanocrystalline films. Nanomaterials.

[51] Khan I., Saeed K., Ali N., Khan I., Zhang B., Sadiq M. (2020). Heterogeneous photodegradation of industrial dyes: An insight to different mechanisms and rate affecting parameters. J. Environ. Chem. Eng..

[52] Asadzadeh Patehkhor H., Fattahi M., Khosravi-Nikou M. (2021). Synthesis and characterization of ternary chitosan–TiO_2_–ZnO over graphene for photocatalytic degradation of tetracycline from pharmaceutical wastewater. Sci. Rep..

[53] Ren Z., Chen F., Wen K., Lu J. (2020). Enhanced photocatalytic activity for tetracyclines degradation with Ag modified g-C3N4 composite under visible light. J. Photochem. Photobiol. A Chem..

[54] Palanivel B., Macadangdang R.R., Hossain M.S., Alharthi F.A., Kumar M., Chang J.-H., Gedi S. (2023). Rare earth (Gd, La) co-doped ZnO nanoflowers for direct sunlight driven photocatalytic activity. J. Rare Earths.

[55] Akhtar J., Tahir M.B., Sagir M., Bamufleh H.S. (2020). Improved photocatalytic performance of Gd and Nd co-doped ZnO nanorods for the degradation of methylene blue. Ceram. Int..

[56] Wang S., Chen Z., Zhao Y., Sun C., Li J. (2021). High photocatalytic activity over starfish-like La-doped ZnO/SiO_2_ photocatalyst for malachite green degradation under visible light. J. Rare Earths.

[57] Pascariu P., Cojocaru C., Samoila P., Olaru N., Bele A., Airinei A. (2021). Novel electrospun membranes based on PVDF fibers embedding lanthanide doped ZnO for adsorption and photocatalytic degradation of dye organic pollutants. Mater. Res. Bull..

[58] Lwin H.M., Zhan W., Song S., Jia F., Zhou J. (2019). Visible-light photocatalytic degradation pathway of tetracycline hydrochloride with cubic structured ZnO/SnO_2_ heterojunction nanocatalyst. Chem. Phys. Lett..

[59] Hosny M., Fawzy M., Eltaweil A.S. (2022). Green synthesis of bimetallic Ag/ZnO@Biohar nanocomposite for photocatalytic degradation of tetracycline, antibacterial and antioxidant activities. Sci. Rep..

[60] Dhiman P., Patial M., Kumar A., Alam M., Naushad M., Sharma G., Vo D.-V.N., Kumar R. (2021). Environmental friendly and robust Mg0.5-xCuxZn0.5Fe_2_O_4_ spinel nanoparticles for visible light driven degradation of Carbamazepine: Band shift driven by dopants. Mater. Lett..

[61] Fan Y., Mo Y., Zhao X., Zuo X., Nan J., Xiao X. (2022). In-situ construction of Bi24O31Br10-decorated self-supported BiOBr microspheres for efficient and selective photocatalytic oxidation of aromatic alcohols to aldehydes under blue LED irradiation. J. Environ. Chem. Eng..

