# Correction: Dhiman et al. Rare Earth Doped ZnO Nanoparticles as Spintronics and Photo Catalyst for Degradation of Pollutants. *Molecules* 2023, *28*, 2838

**DOI:** 10.3390/molecules31020216

**Published:** 2026-01-08

**Authors:** Pooja Dhiman, Garima Rana, Amit Kumar, Elmuez A. Dawi, Gaurav Sharma

**Affiliations:** 1 International Research Centre of Nanotechnology for Himalayan Sustainability (IRCNHS), Shoolini University, Solan 173229, India; 2 Nonlinear Dynamics Research Centre (NDRC), College of Humanities and Science, Ajman University, Ajman P.O. Box 346, United Arab Emirates

## Citation Correction

In the original publication [1], Reference 9 was incorrectly added due to an endnote error. The citation has now been removed, and the order of references was adjusted accordingly.

## Supplementary Materials

The W-H plots in Figure 2 were provided without detailed Supplementary Materials. A correction has been made to explain the figures in Section 2.1. X-ray Diffraction Analysis, in paragraph 4:

W-H plot differentiates the size and strain contribution in peak broadening which also includes instrumental broadening. In observed W-H plot, the less scattering of data points occurs due to negligible 2*θ* variations on rare earth doping at Zn sites. Additionally, the better fit of data points indicates the minimum and uniform lattice strain in samples (Supplementary Materials).

The Supplementary Materials have also been uploaded with the revised manuscript.

The authors state that the scientific conclusions are unaffected. This correction was approved by the Academic Editor. The original publication has also been updated.

## References

[1] Dhiman P., Rana G., Kumar A., Dawi E.A., Sharma G. (2023). Rare Earth Doped ZnO Nanoparticles as Spintronics and Photo Catalyst for Degradation of Pollutants. Molecules.

