# Supplementary figures and images for: Rare Earth Doped ZnO Nanoparticles as Spintronics and Photo Catalyst for Degradation of Pollutants
Source: Molecules. 2023 Mar 21;28(6):2838. doi: 10.3390/molecules28062838 (PMC10058257; doi:10.3390/molecules28062838)

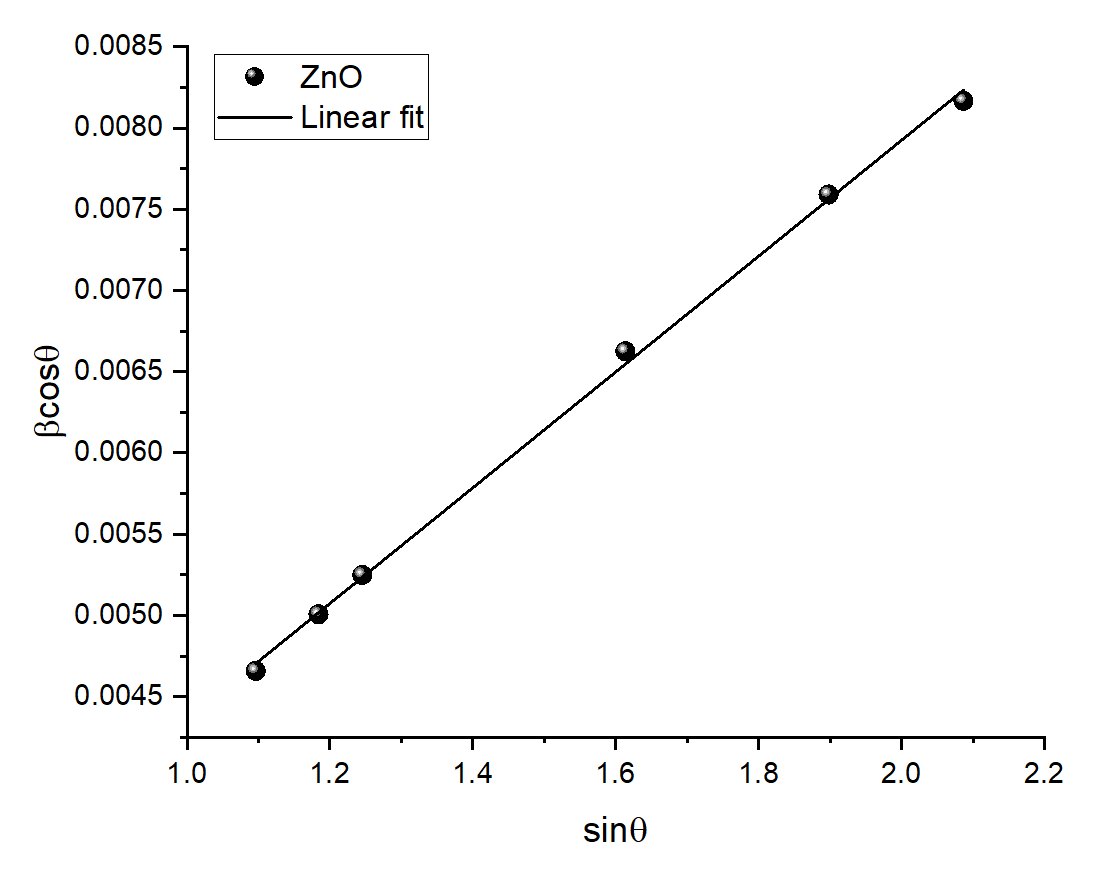

Supplement: Supplementary file 1 [file molecules-28-02838-s001.zip › Graph1-ZnO.png]

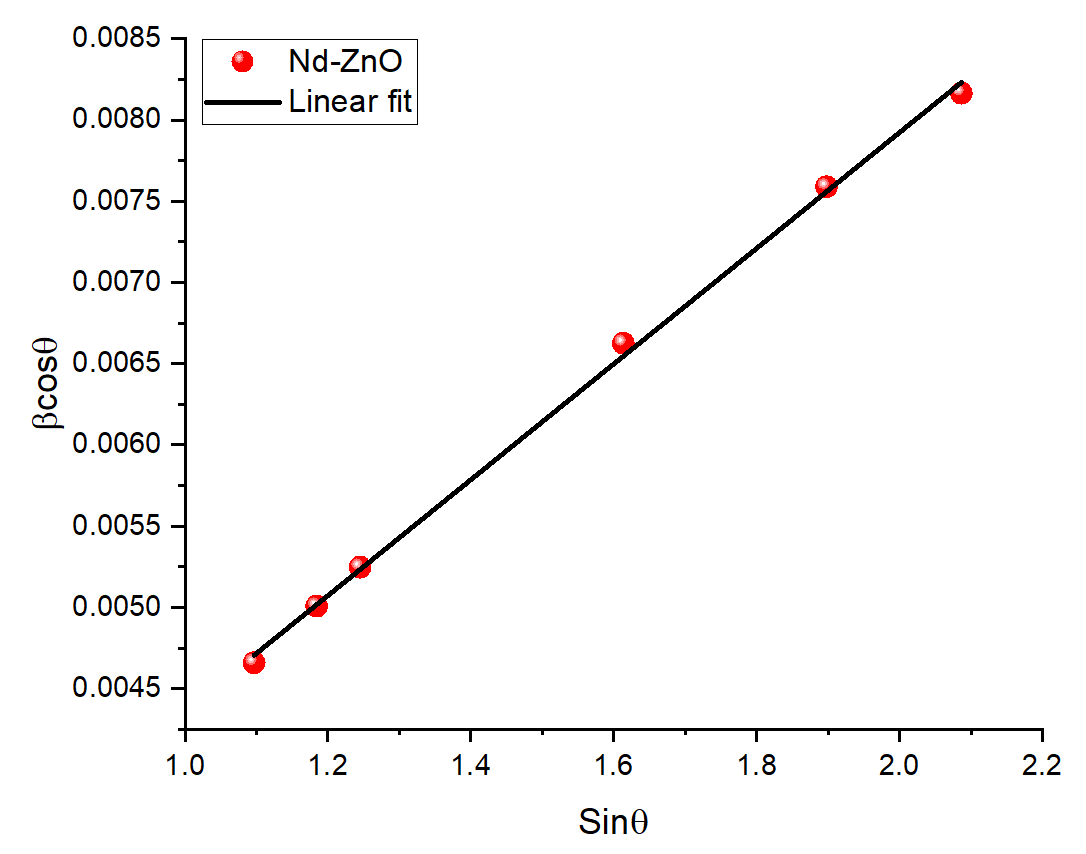

Supplement: Supplementary file 1 [file molecules-28-02838-s001.zip › Graph2-Nd-ZnO.png]

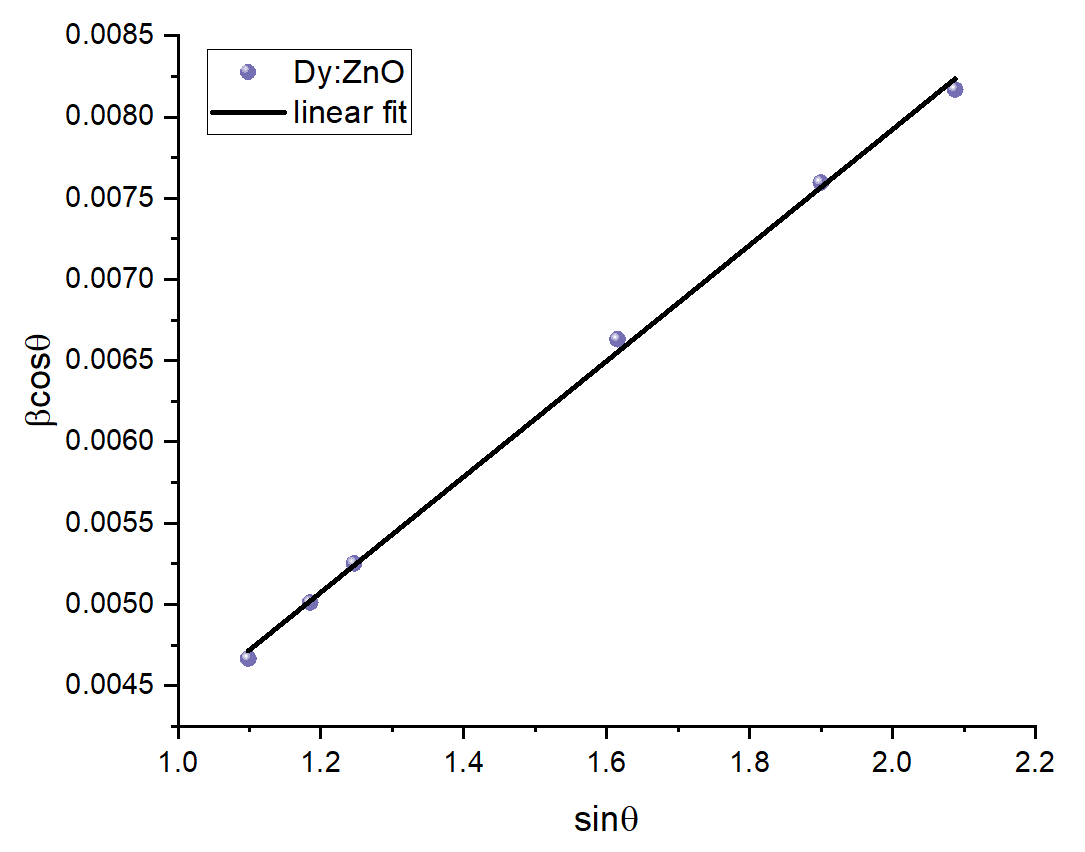

Supplement: Supplementary file 1 [file molecules-28-02838-s001.zip › Graph3-Dy-ZnO.png]

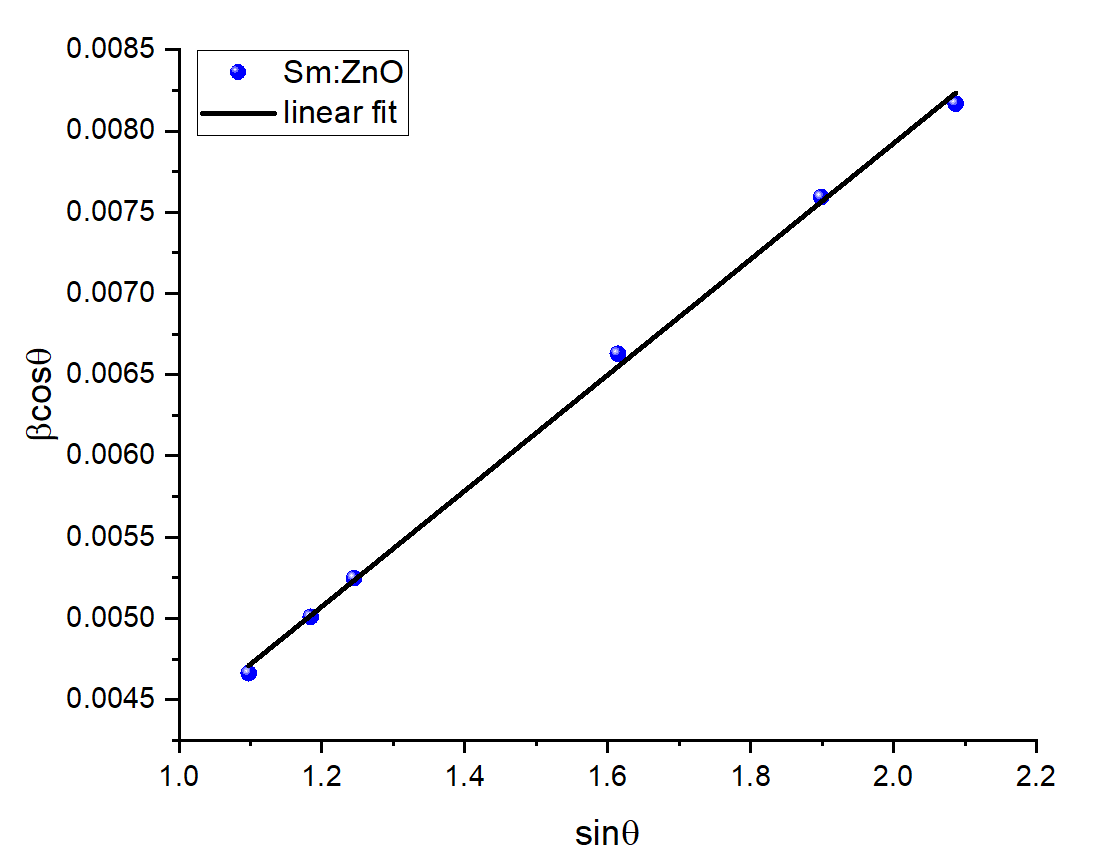

Supplement: Supplementary file 1 [file molecules-28-02838-s001.zip › Graph4-Sm-ZnO.png]
